# Whole Genome Characterization of Lumpy Skin Disease Virus and Bovine Papular Stomatitis Virus Detected in Cattle During the 2024–2025 Outbreaks in Tunisia

**DOI:** 10.3390/v18060622

**Published:** 2026-05-29

**Authors:** Saida Emna Ayari Fakhfakh, Selma Mejri, Makrem Ourabi, Wiem Mouelhi, Hejer Sayari, Soufien Sghaier, Hatem Ouled Ahmed, Aida Tlatli Attia, Tirumala Bharani K. Settypalli, William G. Dundon, Irene Kasindi Meki, Charles Euloge Lamien

**Affiliations:** 1 Laboratoire de Virologie, Institut de la Recherche Vétérinaire de Tunisie, Université Tunis El Manar, Tunis 1002, Tunisia; cherifim2001@yahoo.fr (S.E.A.F.); selma_mejri@yahoo.fr (S.M.); ouledahmedh@yahoo.fr (H.O.A.); attia.aida@yahoo.fr (A.T.A.); 2 LR03ES03 Laboratoire Microorganismes et Bio-Molécules Actives, Faculté des Sciences de Tunis, Université Tunis El Manar, El Manar, Tunis 2092, Tunisia; 3 Commissariat Régional de Développement Agricole de Tozeur, Ministry of Agriculture, Hydraulic Resources and Fisheries, Route de Nafta, Avenue Farhat Hached, Tozeur 2200, Tunisia; ourabimakrem@yahoo.fr; 4 Commissariat Régional de Développement Agricole de Jendouba, Ministry of Agriculture, Hydraulic Resources and Fisheries, BP 588, Assanabil, Route de Tunis, Jendouba 8122, Tunisia; w.ie@hotmail.fr; 5 Commissariat Régional de Développement Agricole de Béja, Ministry of Agriculture, Hydraulic Resources and Fisheries Avenue Habib Bourguiba, Béja 9000, Tunisia; sayarihejer47@gmail.com; 6 Regional Office for Near East and North Africa, Food and Agriculture Organization of the United Nations (FAO), Rue du Lac Winnipeg, Berges du Lac 1, Tunis 1053, Tunisia; sghaiersoufien@yahoo.fr; 7 Animal Production and Health Laboratory, Joint FAO/IAEA Centre of Nuclear Techniques in Food and Agriculture, Department of Nuclear Sciences and Applications, International Atomic Energy Agency, Wagramer Strasse 5, A1400 Vienna, Austria; t.b.k.settypalli@iaea.org (T.B.K.S.); w.dundon@iaea.org (W.G.D.)

**Keywords:** lumpy skin disease virus (LSDV), bovine papular stomatitis virus (BPSV), whole-genome sequencing, Clade 1.2.2, phylogenetic analysis

## Abstract

Lumpy Skin Disease (LSD) is an economically significant viral disease of cattle, widely prevalent across Africa, particularly in sub-Saharan regions. In 2024, Tunisia reported its first outbreak. Understanding the genetic characteristics of lumpy skin disease virus (LSDV) and related poxviruses is critical for surveillance and control. Twenty-nine samples from 26 suspected cases were screened for LSDV using qPCR, followed by a High-Resolution Multiplex Melting (HRM) assay. Three representative samples, two LSDV-positive and one bovine papular stomatitis virus (BPSV)-positive, were subjected to whole-genome sequencing using Pacific Biosciences (PacBio) HiFi long-read technology. Phylogenetic analyses of the LSDV-marker gene RPO30 and complete genomes were performed alongside SNP and InDel profiling. The Tunisian LSDV isolates clustered with Clade 1.2.2 field strains and were 100% identical to each other and to the Italian isolate LSDV_Italy_Sardinia_2025, sharing 99.99% nucleotide identity with LSDV_V281_Nigeria. Although only two LSDV isolates were sequenced which showed no genetic differences, these findings suggest genomic stability within Clade 1.2.2. The Tunisian BPSV isolate showed high similarity (98.15–98.59%) to strains reported in Germany and Switzerland. This study presents the first genetic characterization of LSDV and BPSV in Tunisia, highlighting the importance of accurate differential diagnosis among poxviruses and continuous genomic surveillance to inform control strategies.

## 1. Introduction

Lumpy Skin Disease (LSD) is a highly contagious viral disease affecting cattle, buffaloes and wild ruminants [1]. It was first described in 1929 in Zambia and was restricted to southern and eastern African countries for decades [2,3]. However, since 2012, the disease spread northward and eastward, becoming endemic in the Middle East and Turkey [4,5]. In the recent years, the LSD has significantly expanded its geographical range, reaching new regions in Asia (India, China, Myanmar) [6,7,8], the Balkan peninsula, and Russia [9]. In 2023, North Africa reported its first outbreaks of LSD, beginning in Libya (2023) followed by Algeria in June 2024 and Tunisia in August 2024 [10,11]. In 2025, LSD re-emerged in Europe, with outbreaks confirmed in Italy and France in June, and subsequently in Spain in October [12].

LSD is caused by the lumpy skin disease virus (LSDV), a member of the Poxviridae family, genus *Capripoxvirus* (CaPV), which includes both sheeppox virus (SPV) and goatpox virus (GPV) [13]. Most reported clinical signs of the disease are fever, lachrymation, nasal discharge, salivation, enlarged superficial lymph nodes and nodular lesions on the skin and mucus membrane [14]. LSD can be confused clinically with other diseases causing similar cutaneous clinical signs. Indeed, the bovine papular stomatitis virus (BPSV), the pseudocowpox virus (PCPV) and the orf Virus (ORFV), from the *Parapoxvirus* (PPV) genus can infect cattle, and cause skin lesions that resemble those caused by LSDV [15,16].

The recent LSD outbreaks reported in Tunisia can be chronologically characterized into three periods. Period 1 (Pre-introduction of LSDV): During summer 2024, numerous suspected cases were identified based on clinical signs resembling LSD. Period 2 (First introduction of LSDV): The confirmation of the first LSDV case in Tunisia (August 2024) [11]. Period 3 (Post-introduction of LSDV and commencement of vaccination): Vaccination of livestock began on 7 December 2024.

This study aimed to characterize the complete genomes of LSDV and BPSV associated with the 2024–2025 outbreaks in Tunisia, assess the temporal genomic variation across the three epidemiological periods, and explore the potential epidemiological linkages with regional strains.

## 2. Materials and Methods

### 2.1. Sample Collection and Screening for Poxviruses

The study analyzed samples submitted to the Virology Laboratory of the Institut de la Recherche Vétérinaire de Tunisie between June 2024 and June 2025. The samples originated from privately owned cattle herds located in various regions and suspected of LSDV infection. Sampling was performed by veterinarians with owners’ consent, and no animal experimental procedures were carried out on the animals. A total of 29 samples including anticoagulated whole blood, oral and nasal swabs, swabs from ruptured skin nodules, and scabs, were collected from 26 clinically suspected cattle and analyzed. A detailed sample description and diagnostic outcomes obtained using qPCR and HRM assays are provided in Supplementary File: Table S1.

Viral DNA was extracted from all samples using the DNeasy Blood and Tissue kit (QIAGEN, Hilden, Germany) according to the manufacturer’s instructions. All extracted DNA were tested for the presence of LSDV genome according to a previously described Bowden et al., 2008 qPCR assay [17], using the iQsupermix kit (Bio-Rad, Hercules, CA, USA) and a CFX 96 (Bio-Rad, Hercules, CA, USA) instrument.

DNA amplification for the HRM assay for poxviruses detection (*Orthopoxvirus*, *Capripoxvirus* and *Parapoxvirus*), was performed following a previously described PCR protocol using SsoFast EvaGreen Supermix (Bio-Rad, Hercules, CA, USA) [18]. The HRM assay was performed using the CFX 96 (Bio-Rad, Hercules, CA, USA) instrument, and the dissociation plots were analyzed using the Bio-Rad Precision Melt Analysis Software 1.3. Samples were selected for whole genome sequencing based on viral load (low Cq values), and DNA purity or integrity based on Nanodrop measurements and Agilent Genomic DNA ScreenTape analysis on Tapestation 2200 (Santa Clara, CA, USA).

### 2.2. Clinical Samples Selected for Whole Genome Sequencing

The samples selected for whole genome sequencing (WGS) represented the distinct epidemiological periods and clinical presentations.

**Figure 1:**
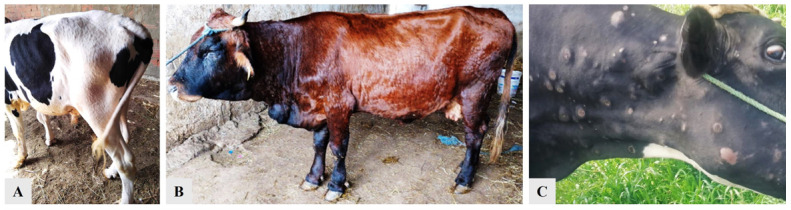
Clinical lesions observed in cattle during three distinct periods of the LSD epidemic in Tunisia between 2024–2025. (**A**): Period 1—Pre-introduction of LSDV, (**B**): Period 2—First introduction of LSDV and (**C**): Period 3—Post-introduction of LSDV and the start of vaccination program.

Sample Tun_24_EB854: A buccal swab was collected in June 2024 from a 13-month-old Holstein male calf from a herd of 15 cattle during Period 1 (Pre-introduction of LSDV). The farm was located in the Imada El Hadhar region of the Tozeur Governorate in southern Tunisia. The calf presented with hyperthermia (41 °C), a mild nodular rash covering the entire body, and papular lesions on the muzzle, lips, and in the oral cavity. The animal died seven days after the onset of symptoms. The DNA from the sample was screened using the Bowden et al. 2018 assay [17] for CaPV detection and the HRM assay for poxviruses detection and differentiation [18]. The HRM analysis confirmed the presence of BPSV (Supplementary File: Table S1). As this was the first diagnosed case of bovine papular stomatitis in Tunisia, and the sample with the lowest Cq value, sample Tun_24_EB854 was selected for molecular characterization by WGS (Supplementary File: Table S1 and Figure 1A).

Sample Tun_24_EN1753: A nasal swab was collected in mid-November 2024 from a local breed cow (>1 year old) on a farm in Guardimaou, northwest Tunisia, near the Algerian boarder, during Period 2 (First introduction of LSDV) that began in August 2024 [11]. The animal exhibited clinical symptoms characteristic of LSD, and the infection was confirmed to be positive for LSDV by both qPCR and HRM analysis (Supplementary File: Table S1 and Figure 1B). This sample was selected for WGS analysis to provide information on the LSDV strain responsible for the initial outbreaks of LSD in Tunisia.

Sample Tun_25_EN477: A nasal swab was collected in February 2025 from a 14-month-old Holstein cow on a farm in the Beja Governorate, northern Tunisia during Period 3 (Post-introduction of LSDV and commencement of vaccination) that began in December 2024 with a live attenuated homologous vaccine. The farm consisted of five adult cattle and is part of the regional dairy basin. The animal was unvaccinated against LSD and clinical signs included lacrimation, nasal discharge, salivation, hyperthermia (41–42 °C), general lethargy and enlargement of the prescapular lymph nodes. qPCR and HRM analysis confirmed LSDV infection (Supplementary File: Table S1 and Figure 1C). This sample was selected for WGS analysis to identify the LSDV strain(s) circulating after the commencement of vaccination.

### 2.3. Whole Genome Sequencing and Analysis

Whole-genome sequencing was performed using the Pacific Biosciences (PacBio) Sequel IIe platform. Approximately, 300 ng of DNA was used for library construction as previously described [19]. After sequencing, the extracted reads were first classified using the Centrifuge classifier v.1.0.4. For LSDV and BPSV genome assembly, CaPV- and PPV-matching reads respectively, were extracted and assembled and assessed as described previously [19].

Open reading frames (ORFs) of the assembled LSDVs and BPSV genomes were predicted using Genome Annotation Transfer Utility (GATU) [20], with LSDV_NW-LW (GenBank accession No. AF409137) as the reference genome for LSDV and BPSV NC_005337 for BPSV. The complete genomes of LSDV_Tunisia_1753NS_2024, LSDV_Tunisia_477NS_2025 and BPSV_Tunisia_854BS_2024, were submitted to GenBank under accession numbers PX778958, PX778959 and PX778960, respectively.

### 2.4. Comparative Genomic and Phylogenetic Analyses

Publicly available LSDV and PPV genomes were retrieved from GenBank for comparative analysis of the Tunisian LSDVs and BPSV genomes. For LSDV, the complete RNA polymerase 30 kDa subunit (RPO30), G-protein-coupled receptor (GPCR) genes, and the partial extracellular enveloped virus (EEV) glycoprotein genes were extracted from the whole genomes and analyzed in MEGA X [21]. A neighbor-joining tree of the complete RPO30 gene was constructed as previously described [19] and visualized with the Interactive Tree of Life (iTOL) v6 tool [22], along with the insertion/deletions in the GPCR or the EEV glycoprotein associated with various LSDV clades. Additionally, 82 LSDV whole genome sequences were aligned using MAFFT v7 [23], and a median-joining network was constructed using PopART program v1.7, with epsilon set to zero, with mutations annotated [24]. Further, Single Nucleotide Polymorphism (SNP)- and Insertion/Deletion (InDel)-based comparative analysis of the LSDVs clustering in the same clade as the Tunisian LSDVs, was performed using SNP-sites v2.5.1 and SNPit v1 and visualized with SNPit [25,26]. For BPSV, a dataset of 33 PPV whole genomes, including 9 BPSV, 3 PCPV and 21 ORFV was retrieved from GenBank and aligned as described for LSDV. A maximum likelihood tree was generated using RAxML v2.0.6 and visualized in iTOL v6.

## 3. Results and Discussion

### 3.1. Preliminary Screening and Detection of BPSV and LSDV Genomes

Among the 26 clinically suspected cattle, 16 animals (61.5%) tested positive for the LSDV genome by qPCR and confirmed by the HRM assay (Supplementary File: Table S1). HRM analysis also revealed the presence of BPSV in two samples. Based on the purity and viral load of the extracted DNA (indicated by low Cq values), three samples; Tun_24_EB854, Tun_24_EN1753 and Tun_25_EN477, were selected for whole-genome sequencing (WGS) and further characterization.

### 3.2. Detection of Parapoxviruses During the Pre-Introduction of LSDV (Period 1)

Sample Tun_24_EB854, was a buccal swab collected during Period 1 (Pre-introduction of LSDV) in a farm located in southern Tunisia. During this same period, a second BPSV outbreak (Tun_24_EB1057), with a high Cq value was detected in Sidi Bouzid, central Tunisia, a major livestock trade hub with high risk of disease transmission. Although BPSV is known to circulate worldwide, it has only been reported recently in Tunisia [11]. The detection in two distinct regions indicates that BPSV may be more widespread in the country than previously recognized.

Clinical signs observed in affected cattle during this period are often confused with other poxviruses infections or unrelated diseases such as cutaneous leucosis, foot and mouth disease, dermatophilosis presenting similar symptoms [27,28,29]. This overlap highlights the diagnostic challenge of differentiating *parapoxvirus* infections from *capripoxvirus* outbreaks, especially during the early stages of an epidemic. This can lead to misdiagnosis or delayed laboratory confirmation and can complicate outbreak response.

Read classification of sample Tun_24_EB854 identified 6264 *parapoxvirus*-specific reads ranging from 634 to 43,643 bp, resulting in a final assembly of 135,907 bp genome, with a mean coverage of 393 ± 72X, and a mapping quality of 59.88. Analysis confirmed the genome recovered from sample Tun_24_EB854 belongs to BPSV (designated BPSV_TUN_854BS_2024) and exhibited high nucleotide identity (98.15–98.59%) to BPSV strains previously reported in Germany and Switzerland and shared 96.51% identity with the reference strain (BPSV_NC_005337) [30,31,32]. Annotation of the BPSV_TUN_854BS_2024 genome using the reference genome BPSV_NC_005337 predicted 131 ORFs, of which; 14 ORFs showed 100% identity, while 104 and 13 ORFs were 90–99.8% and 59.5–89.9% identical to the reference strain, respectively.

**Figure 2:**
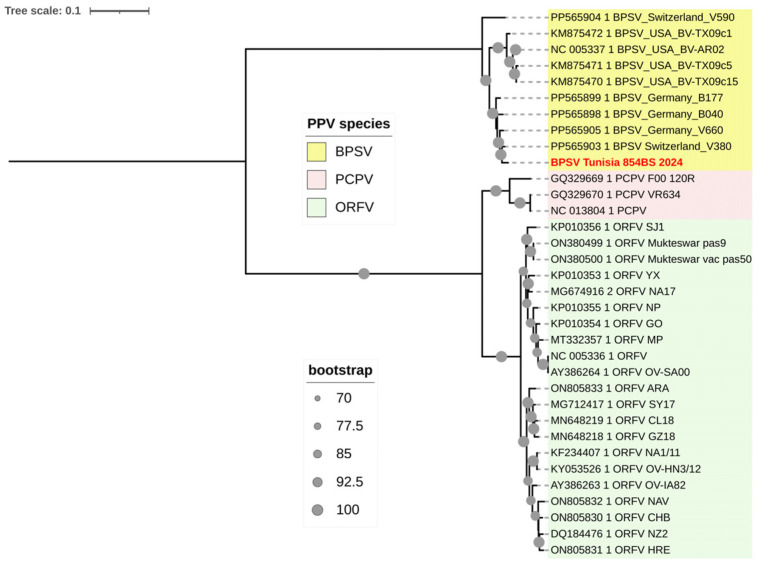
Maximum likelihood tree of 34 *parapoxvirus* (PPV) genomes including BPSV_TUN_854BS_2024 isolate (in red), reconstructed using RAxML v2.0.6 and visualised on iTOL. Bootstrap support values are shown as circles, with circle size proportional to the support value, as shown in the legend scale.

Molecular data on PPVs in Africa remain scarce. Phylogenetic analysis based on the complete genomes of PPVs confirmed that the Tunisian BPSV isolate clusters closely to the isolates from Germany and Switzerland (Figure 2), although this finding may partly reflect the limited availability of sequences for comparison [33,34]. Nevertheless, this study provides the first molecular characterization of BPSV strain identified in Tunisia, contributing valuable genomic data towards understanding of PPV molecular epidemiology and evolution in North Africa [35,36]. The co-circulation of BPSV and LSDV highlights the importance of integrating the differential diagnosis surveillance into national poxvirus monitoring frameworks to strengthen regional epidemiological preparedness.

### 3.3. LSDV Detection During the First Introduction Period (Period 2)

Sample Tun_24_EN1753 was a nasal swab collected in northwest Tunisia near the Algerian border, during Period 2 (First introduction of LSDV). Cattle farming in Tunisia is mainly concentrated in this regional dairy basin, consisting of approximately 228,000 head of cattle, representing half of the national cattle population. This explains why most LSD outbreaks (68%) occurred in the north of the country, while the southern regions have not recorded any LSD cases to date. November and December 2024 marked the peak of the LSD epidemic. Sample Tun_24_EN1753 was collected in the border region, which has been classified as a hot spot due to uncontrolled livestock movements and the high risk of LSDV introduction from Algeria.

### 3.4. LSDV Detection During the Post-Introduction and Early Vaccination Period (Period 3)

Sample Tun_25_EN477 was collected in northern Tunisia during Period 3 (Post-introduction of LSDV and commencement of vaccination). The regional cattle herd was estimated at 11,300 head, half of which remained unvaccinated due to farmer refusal, leading to a spike in LSD cases in the region during this period. Despite a vaccination coverage rate exceeding 80%, refusal by farmers, driven by concerns about vaccine side effects, and poor adherence to biosecurity protocols, was reported in several regions. The rise in LSD cases during this period, raises concerns regarding potential vaccine failure, either due to insufficient vaccine efficacy or genetic mismatch between the vaccine and circulating field strains [37].

#### Samples: Tun_24_EN1753 and Tun_25_EN477 (LSDV)

Read classification of Tun_24_EN1753 sample detected 2630 CaPV-specific reads ranging from 252 to 23,624 bp, producing a final assembly of a 150,892 bp genome bp, with a mean coverage of 83.34X, ensuring comprehensive genome coverage. BLAST analysis confirmed that the genome recovered belongs to LSDV (LSDV_TUN_1753NS_2024). For Tun_25_EN477 sample, 714 CaPV-matching reads ranging from 245 to 35,726 bp yielded a 150,892 bp genome with mean coverage of 31.50X, which was identified as LSDV (LSDV_TUN_477NS_2025).

Both LSDV_TUN_1753NS_2024 and LSDV_TUN_477NS_2025 genomes were 100% identical to each other and to the Italian isolate, LSDV_Italy_Sardinia_2025 (PX222720), including the Inverted Terminal Repeats (ITRs) [12] based on BLAST analysis and shared 99.99% identity with the Nigerian isolate LSDV_V281 [38]. Compared to the LSDV reference genome NW-LW (AF409137), both Tunisian LSDVs showed 99.92% identity. Annotation using GATU with LSDV_NW-LW as a reference genome predicted 158 ORFs, of which; 137 ORFs shared 100%, 16 ORFs shared 95.0–99.9%, 2 ORFs shared 75.0–90.0%, and 3 ORFs shared 2.8–44.2% identity with the LSDV_NW-LW reference strain.

**Figure 3:**
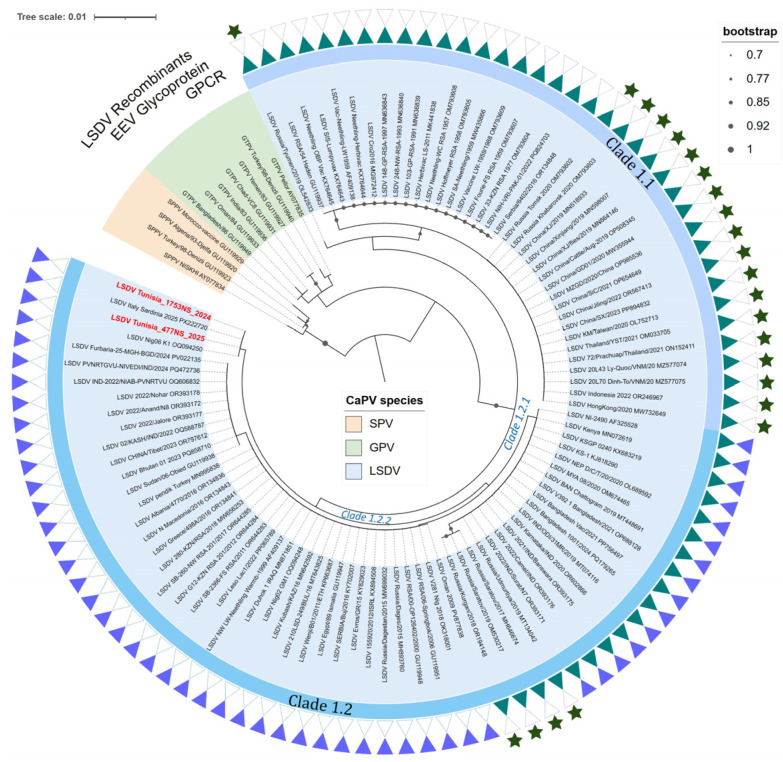
Neighbor-joining tree constructed in MEGA X based on the complete RPO30 gene sequences of 106 CaPVs, using the maximum composite likelihood method with 1000 bootstrap replicates. The tree was visualized on iTOL, along with the presence (filled triangles) or absence (empty triangles) of sequence insertion in the GPCR and the EEV glycoprotein genes. The 2024 and 2025 Tunisian LSDV isolates are in red and known LSDV Recombinants are also shown in green asterisks.

Prior to the recent spread of LSDV in Europe, only a limited number of complete LSDV genomes were available worldwide, limiting geographic representation [39]. For a long time, LSDV characterization relied on partial genomic markers such as RPO30, GPCR, EEV glycoprotein and B22R genes [40]. In this study, phylogenetic analysis based on the complete RPO30 gene sequences, coupled with GPCR and EEV glycoprotein InDel patterns, indicated that the Tunisian LSDV isolates cluster within LSDV Clade 1.2.2 (NW-like LSDVs), grouping with isolates from Sardinia, Italy, and Nigeria (Figure 3) [39].

**Figure 4:**
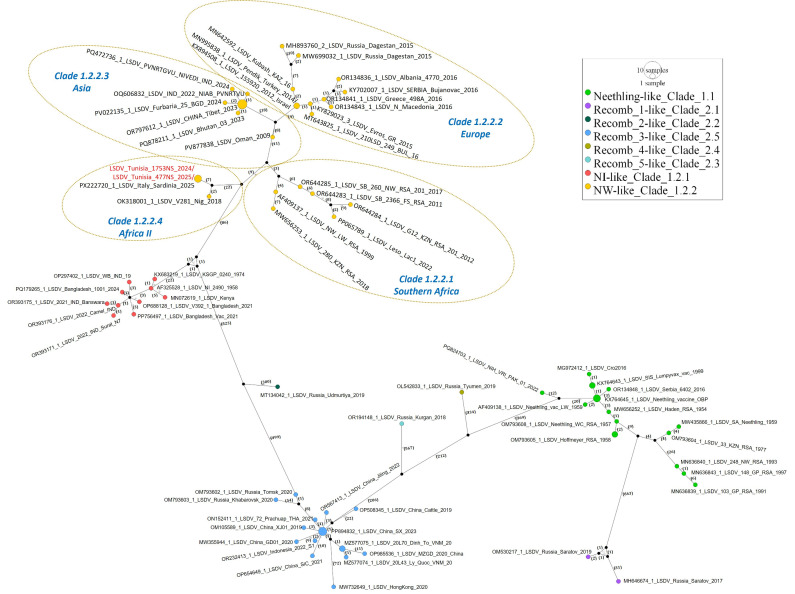
Median-joining network inferred from 82 LSDV whole-genome sequences using PopART program, with epsilon set to zero, showing Tunisian isolates (in red) clustering within NW-like_Clade1.2.2 alongside isolates from Italy and Nigeria. Each circle represents a single LSDV isolate, with circle size proportional to the number of identical genomes. The number of mutations between genomes is indicated in brackets along the connecting branches.

Whole-genome median-joining network analysis further supported their clustering within NW-like Clade 1.2.2, together with isolates from Sardinia, Italy (2025) and Nigeria (2018) (Figure 4) [39].

### 3.5. SNP and InDel Analysis of LSDVs in Clade 1.2.2

**Figure 5:**
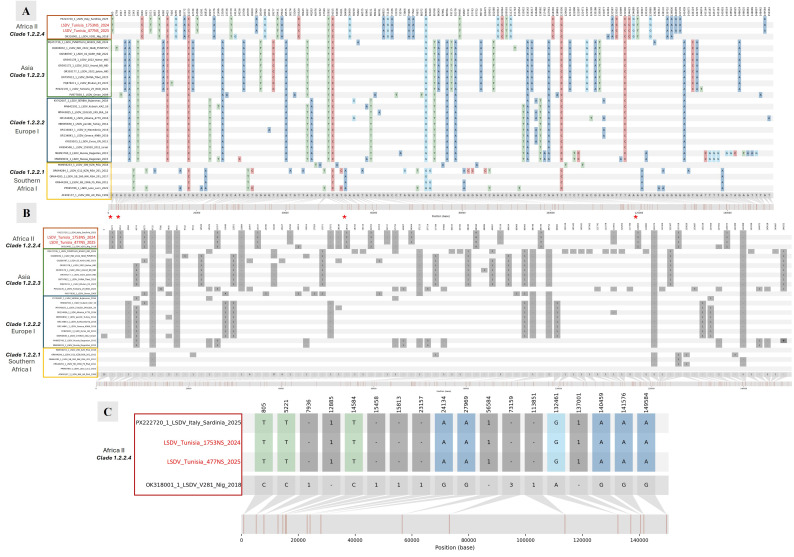
Analysis of SNPs and InDels extracted from the alignment of 31 LSDV whole genomes within the NW_LW_Clade 1.2.2 using SNP-sites and SNPit. Four distinct clusters were identified based (**A**) SNP profile and (**B**) InDel profiles. The unique InDels causing frameshifts and truncation in ORFs in Clade 1.2.2.4 are indicated with an asterisk (**C**) Comparative SNP and InDel profiles of Tunisian LSDV isolates relative to isolates from Italy and Nigeria (Africa II cluster). The Tunisian LSDV isolates are in red.

Comparative analysis of SNPs and InDels was conducted to evaluate the genomic relationships between the LSDV isolates from Tunisia (2024–2025) and conventional non-recombinant LSDV strains circulating in Europe, Africa and Asia. This investigation focused on LSDV Clade 1.2.2 (LSDV NW_LW-like viruses) and identified four distinct sub-clusters, some of which have been previously documented based on whole genome phylogeny [39]. Clade 1.2.2.1 (“Southern Africa I”), comprised isolates from South Africa and Lesotho, clustering with the LSDV NW_LW reference strain. Clade 1.2.2.2 (“Europe I”), consisted of LSDV isolates reported between 2015 and 2018 in Europe. Clade 1.2.2.3 (“Asian”), included isolates from India, China, Bhutan and Bangladesh. Clade 1.2.2.4 (“Africa II”) contained the newly identified Tunisian LSDV isolates, the Italian outbreak isolate (2025), and the Nigerian isolate reported in 2018 (Figure 5A,B) [39].

The two Tunisian LSDVs share identical SNP and InDel profiles with each other and with the Italian isolate, suggesting a possible epidemiological association. Although the dataset represented by the sequenced samples is limited, their 100% genetic identity suggests that the same viral strain has been circulating since the initial incursion, supporting previous findings of slow evolutionary rate of LSDVs in clade 1.2.2 [41,42]. The Nigerian isolate V281, although the closest related strain to the Tunisian LSDVs, differed by 9 SNPs and 10 InDels, suggesting that a similar strain has been circulating in Africa for some time. However, since LSDV Nigeria V281 was isolated following an experimental infection, some of its SNPs and InDels may be artifacts of viral laboratory adaptation, implying the original wild-type virus may have been even more similar to the Tunisian and Italian isolates (Figure 5C) [38].

Notably, Clade 1.2.2.4 exhibited unique genetic features absent from other sub-clusters in Clade 1.2.2, including InDels causing frameshifts and truncations in several ORFs. These include LD006 (interleukin-1 receptor-like protein), LD026b (hypothetical protein) and LD130 (hypothetical protein), showing 44.2%, 2.8% and 8.3% identity to the LSDV_NW-LW (AF409137) reference strain, respectively (Figure 5B). The truncation LD006, a virulence factor involved in modulating host innate immunity, may imply impaired immune evasion, potentially contributing to slower virus replication and reduced disease severity in Clade 1.2.2.4 [43,44]. Such genomic characteristics may partly account for the relatively low morbidity (22%) and mortality (4%) reported during the LSD epidemic in Tunisia, specifically in Period 2 (First introduction of LSDV), where most infected animals reportedly recovered within approximately one month [11]. However, these findings represent a limited snapshot of circulating strains that were sequenced and should not be overgeneralized.

While whole genome phylogeny or network analysis provide a robust evolutionary framework of LSDVs in Clade 1.2.2, the SNP/InDel-based approach applied in this study provides a higher-resolution approach that complements and strengthens phylogenetic inference [39]. First, the approach allows precise differentiation of nearly identical isolates that cluster tightly in phylogenetic trees. Secondly, small relevant mutations such as frameshifts and truncations are captured, which may not be obvious in phylogenetic analysis. Lastly, SNP/InDel profiles independently reveal the same sub-clusters defined by phylogeny, validating the classification proposed by Breman et al. 2023, while offering a mutation-based clustering that can be applied rapidly during outbreak investigations [39]. This demonstrates that SNP/InDel profiling as a valuable complementary tool to phylogenetic analysis, particularly for outbreak-level resolution and functional interpretation of genomic variation.

Targeted amplification of PPV or CaPV genes can differentiate strains, particularly between vaccine and field LSDV strains [40,45,46]. However, such targeted approaches are insufficient for detecting unknown recombinant strains or elucidating LSDV introduction and transmission routes. Whole genome sequencing (WGS) overcomes these limitations by enabling high resolution genomic analysis and improving global understanding of LSDV dynamics [47,48]. In this study, complete genomes of the Tunisian BPSV and LSDV isolates were generated by Pacific Biosciences (PacBio) HiFi long-read sequencing technology, a third-generation platform that represents significant advancement in genomic characterization [49]. Compared to Nanopore Technologies, PacBio HiFi long-read technology generates highly accurate long reads using CCS which is particularly useful for analyzing double-stranded DNA viruses such as poxviruses, which often contain repetitive regions and complex genomic structures [49,50]. Moreover, a key strength of this study is the use of PacBio HiFi long-read technology for direct sequencing of clinical samples, without the time-consuming virus isolation step usually required to concentrate the targeted viral genome [47].

This study has several limitations. First, only three genomes (two LSDV and one BPSV) were successfully sequenced, which restricts representativeness and prevents comprehensive assessment of viral diversity. Second, potential sampling bias may have influenced which cases were available for sequencing, as DNA quality varied across samples. These limitations underscore the need for expanded genomic surveillance across Tunisia and neighboring regions.

## 4. Conclusions

This study presents the first whole genome sequencing and genetic characterization of BPSV and LSDV strains circulating in Tunisia between 2024–2025 LSD outbreak. It presents one of the few genomic reports from North African. Importantly, the investigations described herein were performed before and after the initiation of LSD vaccination, enabling insight into the viral strains circulating during these two epidemiological periods in Tunisia. The two sequenced LSDV isolates showed no detectable genetic differences, suggesting genomic stability, although broader conclusions cannot be drawn due to the small sample size. Expanding the number of complete genomes of LSDV and other poxviruses from different geographic areas (including Tunisia and other north African countries), will be essential to improve the understanding of LSDV and BPSV evolution, trace transmission pathways and inform optimized surveillance and control strategies.

## Data Availability

The original data presented in the study are openly available in the NCBI GenBank under the accession numbers PX778958 to PX778960.

## Supplementary Materials

The following supporting information can be downloaded at: https://www.mdpi.com/article/10.3390/v18060622/s1, Supplementary File Table S1: Description of investigated samples and results of Real-Time PCR and HRM assays. NA*: Not Applicable; DNA quality was assessed based on the viral load (Cq values), and DNA purity/ integrity based on Nanodrop concentration measurements and Agilent Genomic DNA ScreenTape analysis. The samples selected for whole genome sequencing (WGS) representing distinct epidemiological periods, are highlighted in brown, red or green. **Interpretation qPCR Bowden**: Cq value ≤ 38, positive result; Cq value > 40, negative result; 38 ≤ Ct value < 40, Inconclusive result. Note: positive samples are reported as LSDV. **Interpretation HRM POX** Cq value ≤ 38, positive result; Cq value > 40, negative result; 38 ≤ Ct value < 40, Inconclusive result HRM Tm values obtained with CFX 96 (Bio-Rad): [77.2 – 77.4] = LSDV; [75.60 – 75.80] = GPV; [76.20 – 76.40] = SPV; [72.20 – 72.40] = CPXV; [73.00 – 73.20] = CMLV; [81.60 – 81.80] = BPSV; [80.20 – 80.40] = ORFV; [81.20 – 81.40] = PCPV; Note: weak *parapoxvirus* (PPV) signals are reported as PPV but cannot be assigned to any of the three PPV species (BPSV, ORFV, or PCPV).

## Abbreviations

The following abbreviations are used in this manuscript:

LSDV	Lumpy skin disease virus
BPSV	Bovine papular stomatitis virus
CaPV	*Capripoxvirus*
PPV	*Parapoxvirus*
SNP	Single nucleotide polymorphism
InDel	Insertion/Deletion
CCS	Circular consensus sequencing
IGV	Integrative genomics viewer
