# Supplementary material for: Whole Genome Characterization of Lumpy Skin Disease Virus and Bovine Papular Stomatitis Virus Detected in Cattle During the 2024–2025 Outbreaks in Tunisia
Source: Viruses. 2026 May 29;18(6):622. doi: 10.3390/v18060622 (PMC13307912; doi:10.3390/v18060622)
Supplement: Supplementary file 1 [file viruses-18-00622-s001.zip › viruses-4300319-supplementary.pdf]

Supplementary file: Table S1: Description of investigated samples and results of Real-Time PCR and HRM assays

| Sampling Period | Herd/ Animal No | Sample No | Date of collection | Sample ID | Location | Type of Sample | Cq Bowden | Cq HRM | Tm HRM | HRM Results | Observations |
|---|---|---|---|---|---|---|---|---|---|---|---|
| Period 1 : Pre-introduction of LSDV | **1** | **1** | **24/06/24** | **Tun_24_EB854** | **Tozeur (Southwest)** | **Buccal Swab** | **NA*** | **19.25** | **82.00** | **Strong BPSV** | **Whole genome sequencing applied** |
| | | 2 | 24/06/24 | Tun_24_NO854 | Tozeur (Southwest) | Node | NA* | 36.02 | 81.60 | Weak BPSV | Not done |
| | 2 | 3 | 05/07/24 | Tun_24_EB919 | Bizerte (North) | Buccal Swab | NA* | NA* | | | No Poxvirus detected |
| | 3 | 4 | 08/07/24 | Tun_24_EB928 | Kef (Northwest) | Buccal Swab | NA* | NA* | | | No Poxvirus detected |
| | 4 | 5 | 16/07/24 | Tun_24_EB965 | Bizerte (North) | Buccal Swab | NA* | 35.39 | 79.80 | Weak PPV | Low viral load, not possible to genotype and not suitable for Whole genome sequencing |
| | 5 | 6 | 21/07/24 | Tun_24_EB1021 | Kef (Northwest) | Buccal Swab | NA* | 35.07 | 80.80 | Weak PPV | Low viral load, not possible to genotype and not suitable for Whole genome sequencing |
| | 6 | 7 | 29/07/24 | Tun_24_EB1056 | Nabeul (Northeast) | Buccal Swab | NA* | NA* | | | No Poxvirus detected |
| | 7 | 8 | 30/07/24 | Tun_24_EB1057 | Sidi Bouzid (Center) | Buccal Swab | NA* | 31.77 | 81.60 | Medium BPSV | Low viral load, not suitable for Whole genome sequencing |
| | 8 | 9 | 30/07/24 | Tun_24_EB1058 | Jendouba (Northwest) | Buccal Swab | NA* | 34.94 | 80.00 | Weak PPV | Low viral load, not possible to genotype and not suitable for Whole genome sequencing |
| | 9 | 10 | 02/08/24 | Tun_24_ADN01 1077 | Jendouba (Northwest) | Blood | 36.1 | 37.28 | none | none | Weak LSDV, not confirmed by HRM Whole genome sequencing not applied (Cq Bowden >25) |

| | | | | | | | | | | | |
|---|---|---|---|---|---|---|---|---|---|---|---|
| Period 2 : First introduction of LSDV | 10 | 11 | 07/08/24 | Tun_24_EN1133 | Jendouba (Northwest) | Nasal Swab | 27.26 | 35.24 | 77.40 | LSDV | Whole genome sequencing not applied (Cq Bowden >25) |
| | | 12 | 07/08/24 | Tun_24_EB1133 | Jendouba (Northwest) | Buccal Swab | 34.23 | 36.14 | 77.20 | Weak LSDV | Whole genome sequencing not applied (Cq Bowden >25) |
| | | 13 | 07/08/24 | Tun_24_EDTA1133 | Jendouba (Northwest) | Blood | 31.95 | 34.43 | 78.00 | Weak LSDV | Whole genome sequencing not applied (Cq Bowden >25) |
| | 11 | 14 | 13/08/24 | Tun_24_ADN1148 | Kef (Northwest) | Blood | NA* | NA* | | | No Poxvirus detected |
| | 12 | 15 | 26/10/24 | Tun_24_EB1578 | Jendouba (Northwest) | Buccal Swab | 17.18 | 31.70 | 77.20 | LSDV | DNA quality not suitable for whole genome sequencing |
| | 13 | 16 | 26/10/24 | Tun_24_EN1579 | Jendouba (Northwest) | Nasal Swab | 12.59 | 37.62 | 77.00 | LSDV | Low viral load, not suitable for Whole genome sequencing |
| | 14 | 17 | 05/11/24 | Tun_24_EN1721 | Kef (Northwest) | Nasal Swab | 14.93 | 23.68 | 77.4 | LSDV | Low viral load, not suitable for Whole genome sequencing |
| | 15 | 18 | 07/11/24 | Tun_24_EN1725 | Kef (Northwest) | Nasal swab | 18.25 | 31.25 | 77.6 | LSDV | Low viral load, not suitable for Whole genome sequencing |
| | 16 | 19 | 07/11/24 | Tun_24_EN1726 | Kef (Northwest) | Nasal swab | 21.96 | 32.68 | 77.6 | LSDV | Low viral load, not suitable for Whole genome sequencing |
| | 17 | 20 | 08/11/24 | Tun_24_EN1745 | Béja (North) | Nasal swab | 27.41 | 28.72 | 77.2 | LSDV | Low viral load, not suitable for Whole genome sequencing |
| | **18** | **21** | **11/11/24** | **Tun_24_EN1753** | **Jendouba (Northwest)** | **Nasal Swab** | **14.84** | **24.67** | **77.4** | **LSDV** | **Whole genome sequencing applied** |

| | | | | | | | | | | | |
|---|---|---|---|---|---|---|---|---|---|---|---|
| Period 3 : Post-introduction of LSDV and start of vaccination | 19 | 22 | 02/01/25 | Tun_25_EB18 | Béja (North) | Buccal Swab | 23.07 | 32.51 | 77.6 | LSDV | DNA quality not suitable for whole genome sequencing |
| | 20 | 23 | 06/01/25 | Tun_25_EB33 | Ben Arous (Northeast) | Buccal Swab | 20.37 | 31.25 | 77.6 | LSDV | DNA quality not suitable for whole genome sequencing |
| | **21** | **24** | **27/02/25** | **Tun_25_EN477** | **Béja (North)** | **Nasal Swab** | **17.25** | **31.70** | **77.2** | **LSDV** | **Whole genome sequencing applied** |
| | 22 | 25 | 03/03/25 | Tun_25_EB486 | Monastir (Northeast) | Buccal Swab | 21.9 | 32.68 | 77.6 | LSDV | DNA quality not suitable for whole genome sequencing |
| | 23 | 26 | 16/04/25 | Tun_25_EN837 | Zaghouan (Northeast) | Nasal Swab | 22.72 | 31.56 | 77.0 | LSDV | DNA quality not suitable for whole genome sequencing |
| | 24 | 27 | 05/05/25 | Tun_25_EB965 | Manouba (Northeast) | Buccal Swab | 20.74 | 31.96 | 77.2 | LSDV | DNA quality not suitable for whole genome sequencing |
| | 25 | 28 | 06/05/25 | Tun_25_EB966 | Manouba (Northeast) | Buccal Swab | 14.01 | 23.68 | 77.4 | LSDV | DNA quality not suitable for whole genome sequencing |
| | 26 | 29 | 25/06/25 | Tun_25_EB1470 | Béja (North) | Buccal Swab | 18.65 | 31.7 | 77.2 | LSDV | DNA quality not suitable for whole genome sequencing |

NA*: Not Applicable; DNA quality was assessed based on the viral load (Cq values), and DNA purity/ integrity based on Nanodrop concentration measurements and Agilent Genomic DNA ScreenTape analysis.

**Interpretation qPCR Bowden**:

Cq value≤ 38, positive result; Cq value > 40, negative result; 38 ≤ Ct value < 40, Inconclusive result. Note: positive samples are reported as LSDV.

**Interpretation HRM POX**

Cq value ≤ 38, positive result; Cq value > 40, negative result; 38 ≤ Ct value < 40, Inconclusive result

HRM Tm values obtained with CFX 96 (Bio-Rad): [77.2 – 77.4] = LSDV; [75.60 – 75.80] = GPV; [76.20 – 76.40] = SPV; [72.20 – 72.40] = CPXV; [73.00 – 73.20] = CMLV; [81.60 – 81.80] = BPSV; [80.20 – 80.40] = ORFV; [81.20 – 81.40] = PCPV; Note: weak parapoxvirus signals are reported as parapoxvirus (PPV) but cannot be assigned to any of the three PPV species (BPSV, ORFV, or PCPV).
